# Nurses’ experiences of ethical responsibilities of care during the COVID-19 pandemic

**DOI:** 10.1177/09697330211068135

**Published:** 2022-06

**Authors:** Elizabeth Peter, Shan Mohammed, Tieghan Killackey, Jane MacIver, Caroline Variath

**Affiliations:** Lawrence S. Bloomberg Faculty of Nursing, 70379 University of Toronto, Toronto, ON, Canada

**Keywords:** ethics, care, COVID-19, nurses, proximity, moral community

## Abstract

**Background:**

The COVID-19 pandemic has forced rapid and widespread change to standards of patient care and nursing practice, inevitably leading to unprecedented shifts in the moral conditions of nursing work. Less is known about how these challenges have affected nurses’ capacity to meet their ethical responsibilities and what has helped to sustain their efforts to continue to care.

**Research objectives:**

1) To explore nurses’ experiences of striving to fulfill their ethical responsibilities of care during the COVID-19 pandemic and 2) to explore what has fostered nurses’ capacity to fulfill these responsibilities.

**Research Design:**

A generic qualitative approach was used incorporating concepts coming from fundamental features of care.

**Participants:**

Twenty-four Canadian Registered Nurses from a variety of practice settings were interviewed.

**Ethical Considerations:**

After receiving ethics approval, signed informed consent was obtained before participants were interviewed.

**Findings:**

Four themes were identified. 1) Challenges providing good care in response to sudden changes in practice. 2) Tensions in juggling the responsibility to prevent COVID-19 infections with other competing moral responsibilities. 3) Supports to foster nurses’ capacity to meet their caring responsibilities. 4) The preservation of nurses’ moral identity through expressions of gratitude and health improvement.

**Discussion:**

Infection control measures and priorities set in response to the pandemic made at distant population and organizational levels impacted nurses who continued to try to meet the ideals of care in close proximity to patients and their families. Despite the challenges that nurses encountered, the care they received themselves enabled them to continue to care for others. Nurses benefited most from the moral communities they had with their colleagues and occasionally nurse leaders, especially when they were supported in a face-to-face manner.

Conclusion: Moral community can only be sustained if nurses are afforded the working conditions that make it possible for them to support each other.

## Introduction

The COVID-19 pandemic has forced rapid and widespread change to standards of patient care and nursing practice, with nurses having had little opportunity to have their voices heard.^1^ Inevitably, these changes have led to unprecedented shifts in the moral conditions of nursing work. Several studies have explored the ethical implications of the COVID-19 pandemic on nurses’ work. Iheduru-Anderson^1^ and Rezaee^2^ et al. found that nurses have expressed ethical concern with the standard of care that has been provided during the pandemic. In particular, Rezaee^2^ et al. described how spiritual, compassionate, and family-centered care has been poor because of the shift in clinical priorities. Similarly, Sperling^3^ reported on nurses’ perspectives regarding the allocation of scarce resources, with nurses expressing concern for the lack of organizational support. Other studies have focused more on nurses’ emotional responses to these issues, in particular the lack of support for patients because of restrictive visitation policies^4,5^ and the frequent witnessing of suffering and death^6^ of COVID patients.

Less is known, however, about how these challenges are understood through the lens of ethically good care and how they have affected nurses’ capacity to meet their ethical responsibilities. Moreover, little is known about what has helped to sustain nurses’ efforts to continue to care. Without resources and opportunities to adequately care for themselves, nurses will be unable to focus on the needs of others and manage the ethical demands of practice.^7^ More evidence is needed to determine the contexts and types of strategies that are beneficial to support and care for nurses. This understanding is not only essential during the pandemic, but also for future circumstances, so that nurses can continue to provide care at the highest possible standards.

## Purpose

The overall purposes of this research were: 1) to explore nurses’ experiences of striving to fulfill their ethical responsibilities of care during the COVID-19 pandemic and 2) to explore what has fostered nurses’ capacity to fulfill these responsibilities.

## Theoretical Underpinnings

The work of Vanlaere and Gastmans^8^ was used as a theoretical lens. They stipulate four fundamental features of care drawing on related work in virtue, care, nursing, and feminist ethics which are described below:

(1) “Care is simultaneously a way of life and an ethical task” (p.16)^8^

As a way of life, care is fundamental, often considered instinctual or natural, involving all human activities that better the human condition, including self-care.^8^ As an ethical task or responsibility, care must be other-regarding in that attention and involvement must turn to the needs of others. The goal of nursing activity is “the promotion of the well-being of the patient by providing good care in the wider meaning of the word—that is, on the physical as well as the psychological, relational, social, moral, and spiritual levels” (p.45).^7^ In doing so, nurses enable patients to maintain a sense of identity by preserving their association to the social and cultural world despite the depersonalization of illness and the routinization of institutional work.^8,9^ Nurses’ ethical task of caring for persons is not only necessary for those who are in close proximity but is also required for unknown and future others through health policy.^8^ When patients are immediate, however, the closeness to pain, suffering, and vulnerability generates strong normative intuitions in nurses to care and attend to their needs.^10^

(2) “Care is a practice by which attitude and activity go hand-in-hand” (p.20)^8^

Care involves an attitude of “caring about” which refers to the emotions and attentiveness of the caregiver along with taking responsibility for caring activities in response to another’s needs, that is, “caring for.”^8^ Attentiveness requires that caregivers have had their own needs sufficiently met so that they can put aside their own concerns and goals to recognize others’ needs.^7,11^ The activity of care involves “*face-to-face* interactions between both persons” (p.21),^9^ that is, in order to be considered good care, must be competent and recognized by the care recipient as effective.^11^

(3) “Care is reciprocal” (p.24)^8^

Not only are care recipients dependent on caregivers to have their needs met, caregivers also are dependent on care recipients to maintain their moral identity.^9,10^ In nursing, when patients express gratitude, display improvement or confidence, or show signs of being physically comforted, they provide nurses with a sense that they are good nurses.^10^ In this way, “patients can *complete* the caring presence of the nurse” (p. 51).^7^

4) “Care is meaning-giving” (p.27)^8^

Ultimately, with reciprocity, nurses can find life fulfillment and meaning in providing care. However, nurses can experience stress and meaninglessness with limited personal contact with patients is possible because under these circumstances they cannot receive patients’ responses.^8^

## Methodology

We chose a generic qualitative approach because established methodologies could not provide us with the flexibility needed^12^ to integrate ethical theory throughout the research process to clearly conceptualize nurses’ ethical responsibilities. This methodology encourages flexibility to tailor methods and the use of appropriate techniques from existing qualitative methodologies to best answer research questions.^12^ To maintain coherence, we chose to locate this work within an interpretivist paradigm where meanings are understood to be generated through social interaction.^13^ Vanlaere and Gastmans’^8^ work is consistent with the characteristics of this paradigm because of its emphasis on nurse-patient relationships and the meaning of care.

After receiving ethics approval from the University of Toronto’s Health Sciences Research Ethics Board, participants were recruited using purposive sampling through the Lawrence S. Bloomberg Faculty of Nursing’s graduate student list serves and Facebook groups, the university’s web-based learning management system, and snowball sampling. Inclusion criteria included: (1) Registered Nurse (RN); (2) experience providing direct clinical care to someone with, or suspicion of, COVID-19 infection; and (3) English fluency because of the language skills of the researchers. One exclusion criterion included: (1) a current student of any of the researchers. Because we used passive recruitment strategies, we do not have insight into why some nurses chose not to participate. To obtain a balanced appreciation of the contextual influences on care, we recruited a heterogeneous sample of 24 participants from 12 hospitals and three community/public health organizations from six cities in the province of Ontario and from one city in the province of Nova Scotia, Canada. See Table 1 for participants’ years of experiences and areas of practice. This sample size allowed us to achieve data saturation and develop a rich understanding of nurses’ care in the context of the pandemic.^14^

**Table 1.** Participant characteristics.

Participant number	Speciality/Setting	Years of experience
1	Intensive care unit	2 to 4
2	Resource team	2 to 4
3	Community	2 to 4
4	Resource team	2 to 4
5	Labor and delivery	2 to 4
6	Community	10 to 14
7	Mental health	2 to 4
8	COVID assessment center	2 to 4
9	Emergency department	10 to 14
10	Mental health	2 to 4
11	Cardiac care unit	2 to 4
12	COVID unit	5 to 9
13	Community	15 to 20
14	Intensive care unit	2 to 4
15	Respiratory	15 to 20
16	Pediatric intensive care unit	5 to 9
17	Emergency department	5 to 9
18	Intensive care unit	5 to 9
19	Internal medicine	15 to 20
20	Intensive care unit	5 to 9
21	Emergency department	2 to 4
22	Emergency department	5 to 9
23	Intensive care unit	15 to 20
24	COVID assessment center	2 to 4

We conducted semi-structured, audio-recorded interviews of approximately one-hour each using Microsoft Teams because of COVID-19 restrictions during the spring and summer of 2020. Interviews were a suitable form of data collection because they elicited participants’ reflections of their care during the pandemic. Participants were asked about their work and educational background; how the pandemic was affecting their care; the ethical challenges and sources of stress they were encountering; what helped or would help them cope; the influence of colleagues, managers, and the public. The audiotapes were transcribed by a professional transcriptionist, de-identified, and then stored on a secure server.

## Ethical Considerations

An informed consent document was signed by the participants, which described the study, the risks and benefits, the opportunity to withdraw, data security, and measures to maintain confidentiality, including the use of participant numbers as opposed to names. All recruited participants completed the study. As a form of appreciation, participants received a $30 electronic gift card of their choice at three different businesses after their interviews. Because this amount is less than the hourly rate for nurses in Canada, it was not considered coercive.

## Data Analysis

Data analysis was a collaborative and iterative process which occurred independently and collaboratively during regular research team meetings of the first four authors. Nvivo was used to manage the data and facilitate the analysis. Initially, codes were identified in an iterative fashion using constant comparison and staying close to the words of the participants. Later deductive techniques employing the four fundamental features of care ethics to add depth, contextualization, and theorization to the analysis.^15,16^ Tentative themes were identified and then we went back and forth between these themes, care ethics, and the research questions until there was coherence and agreement regarding the naming and key analytical characteristics of the themes.

Trustworthiness was maintained by being attuned to the interrelationships between the perspectives of the participants and our interpretations of them. To further promote trustworthiness, we closely followed the data collection strategies, ethical guidelines, and analytical procedures.^17^ We engaged in reflexivity by being aware of the influence we had on our interactions with participants and our process of analysis.^18^ We kept reflexive notes of our reactions, assumptions, and insights that reflected our own identities as nurses, faculty members, researchers, and people impacted by COVID-19.^17^

## Results

Our analysis yielded four themes that conceptualize the experiences of nurses striving to fulfill their ethical responsibilities of care and describe what fostered their capacity to fulfill their ethical responsibilities.

(1) Challenges providing good care in response to sudden changes in practice.

To manage the rapidly evolving COVID-19 crisis, changes to nursing staffing models were instituted very quickly and hospital beds were emptied to accommodate patients. These changes to practice reflect organizational and governmental decisions that were made at a distance and generally without nurses’ involvement but had great impact on their frontline work in close proximity to patients. These changes presented barriers to good care from the perspective of nurses in several ways.

One nurse described the impact of the sudden changes this way:

Everything kept changing so rapidly, there was no time to acclimatize to any kind of new norm. And that invisible labour of constantly renegotiating, adjusting, moving in that capacity of resilience sits differently for a lot of people, but I think it’s part of that spectrum of invisible labour that really isn’t qualified.(7)

Due to changes to nurse staffing models, such as the redeployment of nurses to the intensive care unit (ICU) to accommodate the influx of COVID-19 patients, nurses had to contend with working in new areas of practice without sufficient staffing or clinical resources. As a result, not all nurses believed they were adequately competent to care for these patients.

One participant said:

Because I’m not an ICU Nurse I’m not supposed to be caring for these patients independently, but I’ve had agency nurses that didn’t look over everything that I was doing, which – I don’t mean like in an intense way, but I’m not an ICU nurse, so I think that you should review my rhythm strip, or just take a peek at the patient to make sure they look okay to you.(18)

Freeing hospital beds to accommodate COVID patients had implications for nurses in the community and in ICUs. A community nurse identified the distress they experienced when the shelter system was overwhelmed with people rapidly discharged from acute care, reflecting the further marginalization of people in need of shelters:

The hospital system is trying to get people out…So now that everyone is kind of mass dumped into the shelter system, so just trying to figure that out. Because it is challenging dealing with the influx, and I’m only one person. I can only do so much.(3)

In addition to quick discharge from hospital, participants described their concerns regarding the withdrawal of non-COVID related treatment from vulnerable patients to make room for COVID patients:

The people who have been here for like months and months, they were very strongly pushed for the withdrawal, and they’ve contacted their families, and over the weekend, just like, people started going, and I couldn’t believe that... It seemed like there was a handful that they kind of just decided, okay, we’re supporting this person, but we need the beds…The nursing culture, I think, is very happy to keep a chronic patient. Like, there’s no harm in him being here, or them being there.(2)

Through this excerpt, this nurse reveals that the usual attentiveness along with the extensive involvement with the family when treatment withdrawal is performed was forgone to make beds available in a rapidly evolving crisis.

(2) Tensions in juggling the responsibility to prevent COVID-19 infections with other competing moral responsibilities

Nurses experienced the tension between needing to meet their usual caring responsibilities and simultaneously needing to prevent the spread of COVID-19. The most common way nurses experienced this tension was in relation to visitation policies, the requirement to don and doff PPE, the responsibility to ensure that patients adhered to infection control measures, and the reduction of community services.

Because of policies that restricted the presence of family in hospitals, nurses could not reach their ideals of family-centered care and beyond this, often held the responsibility of enforcing these policies, despite not having participated in their creation. A nurse, referring to a laboring patient, stated:

She had had five previous losses. And she was having, like, had finally carried a baby to term and developed a fever. And I had to ask her husband to leave. Can you imagine?.. It’s just awful. Just awful. And I think everybody was really horrified of having to be put in those positions of asking people to leave, or people come into triage, and you have to tell them that their husband can’t come with them.(5)

Nurses’ proximity to their patients generated a strong caring attitude when COVID measures disrupted patients’ face-to-face contact with others. They spoke of patients’ loneliness(23) and how horrid it was to watch patients in isolation without the emotional and physical support of family.(15) Witnessing and trying to support dying patients without family present was experienced as very distressing. One participant said:

Usually when a patient is imminently dying, the family would be able to at least hold their hand, even if the patients themselves weren’t conscious, just that physical touch I feel like was immensely comforting to the family. But that’s not possible now.(1)

Nevertheless, nurses did their best to facilitate patients’ relationships with their families, despite the visitation policies. Some found ways to have family present when patients were imminently dying, and many provided tablets and other virtual means for families to connect. For example, one participant said: “We have tried to introduce iPad communication, daily video chats, anything we can to let that face time happen between patients and their family members. And I think it helps quite a lot.”(15)

Although PPE was necessary, and nurses understood the importance of it, it acted as a barrier to nurses’ moral actions by inhibiting nurse–patient relationships and slowing down their ability to care for patients. Nurses spoke of the time-consuming nature of donning PPE even in emergent situations, such as the one described below:

The patient is coding and rapidly de-sating, but you have people gowning up outside, and it takes a while to put on all your PPE, right? So, you’ll be watching the sat drifting down, and you’re trying to put on your N95 as fast as humanly possible.(20)

Others spoke of PPE interfering with face-to-face relationships with patients. For example, one participant said:

I feel like a robot and very distant from my patients. That whole, you know, ‘treat the patient holistically, person-mind-soul-spirit’ I think that’s gone by the wayside. I feel like that important aspect of providing care and, you know, touching a patient’s shoulder with a bare hand, you know, that human touch is lost…And I feel terrible because the patients can’t see my face because I’ve got a face mask on, a face shield on, a yellow gown, scrubs, gloves, and… it sort of removes yourself from the patient, you know? And then you always have to say to yourself, “this is a human being and not a body.(19)

In mental health settings, unique circumstances arose in which nurses were required to restrain patients to prevent them from spreading COVID-19 which resulted in patients not being able to freely move about and a disruption in nurses’ caring relationships with their patients. One participant stated:

To mitigate risk while also doing the least restraint, we’ve had to lock someone’s door essentially to mitigate them coming out, because they’re just not in a place where they can cognitively understand the risk that is imposed upon them, other people, the staff. At some points, we’ve had to do chemical restraints as well until we’re able to safely ensure that that person is not COVID positive.(7)

Community services were also reduced to stem the spread of COVID-19 resulting in nurses not having face-to-face contact or the resources they required to meet clients’ needs. One nurse described the challenge of not being able to conduct well baby visits(13) and another said:

If you look at the homeless community here in X, for example, all of the services that our homeless community would typically access from a drop-in basis are gone. And so now, I mean, I am literally the only street outreach nurse in X at the moment. And it’s a huge amount of pressure…I feel like there’s a huge need and I feel like I can’t provide the kind of care that I want to be providing because there’s only one of me and because the need is just so high.(6)

(3) Supports to foster nurses’ capacity to meet their caring responsibilities.

Participants shared a range of experiences with respect to supports offered to nurses by their clinical organization. Some participants described an oppressive organizational context that they perceived to be characterized by a lack of trust, concern, and transparency which contributed to their sense of being unsupported, while others provided examples in which organizational leaders demonstrated care and support.

One participant described nurses having no input into the decision to make their unit a COVID unit, leaving them unprepared, and potentially lacking in competence, to care for this patient population. They said:

All of us were pretty blindsided by this decision. And I think also there was no input from frontline staff whatsoever when they made this choice. And that was difficult too…I didn’t come to this work to be told “You have no choice. You’re now doing something that is so far from what you would normally be doing.(10)

A lack of care and concern for nurses also manifested itself through administration’s treatment of nurses who were sick with COVID-19 and through their limited in-person contact. One nurse expressed feeling devalued by the organization:

I perhaps feel a bit more disposable than I did at the beginning of this. I chose the COVID unit because I thought that’d be the safest place, but in the end, I still got COVID…I just feel like they didn’t care as much as I wish they would have is the issue. In the end, at the end of the day, the nurses were still the ones showing up for every shift, every night shift, every weekend; when everyone else went home, no one else were in those rooms when those aerosol-generating procedures were happening. Or when there was a COVID positive patient, the APN (Advanced Practice Nurse) would never step in the room, the manager never needed to go in the room.(18)

The lack of face-to-face interaction by management further created an atmosphere that bred a sense of a lack of support and care for nurses. For example, one participant said:

The director for that (nursing) governing body never even set foot really on a unit – any unit... I was struggling with just being on a COVID unit and working alongside others every day, trying to support them and lead them through, you know, what everyone’s been calling an unprecedented time. Meanwhile, this large body that represents nursing and nursing professional practice was absent and disconnected, not on the same page. That was very, very upsetting and frustrating for me.(12)

In contrast, others spoke of their managers in very positive ways with respect to their support, openness, and presence:

So, having that reflective practice has been a really good support because I’m able to kind of dump everything out during that hour and get a lot of feedback and support back, too. My supervisor is really good at filling up our cup.(13)

Another said,

I actually felt as though the relationship (between frontline staff and leadership) was quite good because we felt very supported. We always had a leadership team member pretty much there every day, even on weekends, when it was really bad. So, then we felt super supported.(22)

Receiving professional therapy, which was occasionally provided by the organization, was met with mixed responses. Some described this support as valuable, whereas others expressed hesitation because the therapists were too far away from the realities of their work. For example, one participant said:

The organization offered, the psychologists were offering one-on-one support through over the phone, virtually, as well as EAP (Employee Assistance Program). But staff were very reluctant, and, again, it felt like that whole layer of disconnect. Like, I don’t want to talk to someone on the phone who can’t possibly appreciate what I am going through and what I am living through right now.(12)

The strongest and most common source of support recognized by nurses was through their nursing colleagues, particularly in the context of face-to-face interactions. Participants found it particularly helpful if their colleagues had similar experiences and could recognize and validate their painful experiences. One participant noted:

... your colleagues really are there for you. That kind of stuff has been a great source of support for myself, and I hope for my colleagues… that have been directly involved in the most distressing situations. I feel like speaking to that person and just kind of validating, “yeah, that was terrible. Yeah, that was an awful situation.” Having those feelings validated I think has been probably one thing that has assisted me.(9)

Another said: “Having that camaraderie and leveraging that camaraderie with my colleagues who understand and are in similar shoes has been very helpful.”(11)

Another spoke of physical touch that was offered by their colleagues in response to their distress. They said:

Strangely, since COVID the level of just being collegial it feels like it’s gone up. Because I feel like people need to support each other more… But realistically we’re a unit that is fairly tight-knit and we need to come together to help each other out a lot. And we hug each other, and we pat each other on the back, and we show each other – we’re like a large group of women who are all kinds of ages, and it’s not uncommon for someone to come and rub your shoulders while you’re on the computer or for someone to give you a hug. Some of the older nurses will come and just pat you on the head.(5)

Similarly, but less common, participants spoke of the importance of the interprofessional team who also offered support and recognized the difficulties they were facing. One participant said:

I feel that there’s a good, strong sense of the team, that it’s imperative that we work together, that we are all facing these risks. I think there’s a recognition of the type of specific risk and difficulties for each profession that their inter-professional colleagues are recognizing.(1)

Underscoring the importance of proximity in offering support, another said: “the clinical lead actually came inside the room with me at that time.”(11)

Nurses also relied on self-care activities to help sustain their capacity to continue to meet their caring responsibilities. These included, “binge watching a lot of TV, playing video games, online shopping,”(24) “exercising more,”(21) “running a lot,”(22) “journaling,”(16) and “seeing my partner, going for a walk, feeling safe, just being outside, talking to my family, anything that normally makes me feel better.”(2) Others spoke of using a “mindfulness meditation type app”(9) and prayer: “I believe in God. So, a little prayer when you are sad helps. Whenever I get a little time, I close my eyes and I just pray to God.”(23)

(4) The preservation of nurses’ moral identity through expressions of gratitude and health improvement

Patients’ responses either through the improvement of their health or expressions of gratitude completed the caring process, thereby helping to sustain nurses’ moral identity as illustrated in the following quotes:

And then when she woke up, all of a sudden, and I was the nurse. Oh my God, I was jumping! So happy that the tears were coming out of my eyes. She was nodding appropriately. She was squeezing my hand… I need some happiness from my patients, from my work. It’s not that I just need happiness from my family or from my personal life. I work half of the time here. Twelve hours I work here, right?(23)

Another stated: “We had one patient who told me, “Oh, you’re making me feel so calm…” He was like, “Oh, I’m so glad you’re here.”(9) Participant(17) simply described the importance of appreciation in sustaining her by saying: “Honestly, every time someone said thank-you so much for what you do.”

Although nurses valued the gratitude of patients, the public’s response, such as pot banging, cheering outside hospitals, and statements of thanks, was received with mixed reactions. At times, it was appreciated, but other times, it was met with skepticism. For example, for many nurses being called a hero by the public was not suitable as an ideal to internalize as constitutive of their moral identity and not representative of their work during the pandemic. One participant said that the hero worship would do little to yield long-standing change:

I have the same patient ratio, I’m doing the same work, and I’m giving the same care that I do every day of the year… and when this is all over, I’m going to be doing the same kind of care when all the hero stuff goes away. I’m still going to be working this hard.(14)

In contrast, another said:

And all over the radio when you listen to it, it’s like, “nurses and doctors, thank you so much. You’re the heroes.” And I’m like, no one’s ever called me that! No one’s ever – I feel like any time I say that I’m a nurse, there’s always a handful of people who are like, “oh, you’re just a nurse?” Or something like that. And now it’s like, “oh my gosh, you’re the hero!” I’m like, huh there you go.(2)

With respect to corporate contributions, participants expressed ambivalence because it was not always seen as an authentic expression of gratitude. For example, one nurse said:

I personally started to feel like it became a bit more of a performative action, like a virtue signaling of companies just kind of jumping in to say that they did something for nurses or frontline heroes to make their company kind of look better.(4)

## Discussion

The results of this research point to less commonly identified ways that the provision of good care has been compromised during the COVID-19 pandemic. Nurses experienced challenges in fulfilling their moral responsibilities and enacting their moral identities because of public health measures that led to restrictions in practice, such as visitation policies. The diminishing of nurses’ moral agency in their caring work and their perception of not being supported were sustained by organizational cultures that lacked transparency and provided few opportunities for frontline nurses to participate in decision-making in relation to public health measures and new organizational priorities. Nevertheless, some organizational, collegial, and personal strategies helped nurses to provide good care.

Many of the challenges nurses in our study experienced were a result of public health measures that were translated into organizational policies by senior administrative leaders. These powerful measures, which often take the form of government policies, frequently impede personal liberties. Typically, they are understood to create ethical tensions or dilemmas between the collective good and the rights of individuals, yet they are not necessarily without ethical justification.^19^ In the context of a pandemic, these measures, along with prioritization decisions, are used to maximize the number of lives saved and protect the public from harm.^19^

While one of the ethical tasks of caring is understood to involve health policies that address the needs of people who are not in close proximity,^8^ our participants were not in a position to make choices about how these measures would, or would not, be imposed. Instead, most our participants experienced the moral impact of these policies in close proximity to patients and their families, not at the level of an organization or the distant level of population health. Insights from the enduring work of Gilligan^20^ and Nortvedt^10^ on the ethic of care speak to how the moral world can be encountered differently when people come across ethical concerns that involve people in close proximity versus those at a distance, such as members of the public we have not met. Concerns involving those in close proximity generally create strong moral motivations to respond in those nearby^10^ with the unique characteristics and circumstances of people involved often being viewed as more important than abstract rules and principles,^20^ such as those articulated in policies. While public health measures can be ethically justifiable in the context of a pandemic and the participants in our study adhered to them as best as they could, overall, they experienced them as challenging when the immediate situations they found themselves in demanded caring responses they could not fully provide.

Therefore, it is not surprising that participants found visitor restrictions to be a source of ethical concern given both their lack of input into organizational decisions and their proximity to patients. The witnessing of patients dying without family present was viewed as a compromising good care given the centrality of family-focused care, which highlights the importance of family present at death to humanize and support the dying. Because nurses’ moral identities are sustained by holding the identities of vulnerable people,^9^ often with the inclusion of family, the participants’ inability to be adequately present with dying patients was threatening to their moral identities.^21^

In addition, ethical decision-making during a pandemic should be guided by procedural values, such as transparency and inclusiveness, so that decision-making is open to scrutiny, and stakeholders have opportunities to be engaged in the decision-making process.^19^ Some participants explicitly expressed concern that there was little transparency or requests for their input into how decisions were being made with respect to the pandemic response in their organizations, contributing to their experience of restricted moral agency. Nurses frequently found themselves without the needed expertise and resources to practice in a way that supported the provision of good care. While our findings are novel because they pertain mainly to nurses’ frequent lack of input into infection control measures and organizational priorities during a pandemic, the organizational context in which they found themselves in is very familiar, harkening back to Jameton’s identification of the “institutional constraints” (p.6)^22^ that frequently shape nurses’ moral lives.

In terms of fostering nurses’ capacity to meet their caring responsibilities, our participants pointed to several organizational, collegial, and self-care strategies. Participants found that a supportive moral community, that is, one that fosters moral dialog, reflection, mutual respect, and collective moral support, to be helpful.^23,24^ They consistently spoke of the importance of engaging in dialog with their colleagues, especially when these conversations occurred with those who shared their own experiences and concerns. Moral communities are places where people can speak openly, and critically reflect on, their responsibilities, values, and concerns as members of a particular community. They encourage open and respectful moral discourse from all their members.^24^ These participants emphasized the significance of validation, reflection, collegiality, and occasionally physical touch, all attributes of a moral community in which people are cared about and cared for. Face-to-face discussions with colleagues who shared their experiences were viewed very positively, as opposed to those with managers or therapists who would not, or could not, make themselves available in person and, therefore, could not fully demonstrate attentiveness and understanding. Moral communities were therefore linked to the notion of proximity to mitigate the effects of a work environment where physical barriers, isolation, and distance were required to curb transmission.

Other strategies were also identified to have a positive impact on fostering nurses’ capacity to care. For example, self-care strategies including exercise, meditation, and distraction were found to be helpful to participants. In addition, participants found that seeing patient improvement and expressions of gratitude from patients was helpful. Witnessing or receiving messages about patients’ recovery has the potential to bolster nurses’ moral identity as good people who can make a difference in peoples’ lives.^9^ In essence, these patients completed the caring process.^7^ It is essential, that this finding is viewed with caution, however, because not all patients improve and not all patients can, or should be expected to, express gratitude.^9^ Moreover, in contrast to other studies,^25,26^ we did not find that these nurses spoke of finding meaning in their work.

The responses of the public and corporate support were met with ambivalence. While some nurses experienced a sense of being valued and empowered, others were quite skeptical suggesting this adulation might be short-lived, a form of virtue signaling, and no substitute for good working conditions and remuneration. The nurse as hero discourse was also questioned as a positive response because heroes, like angels and saints, are expected to engage in supererogatory acts. Hero worship may spawn a kind of moral ideal that is impossibly high and potentially oppressive, particularly during a pandemic with a shortage of resources. The hero discourse is also problematic because it normalizes nurses’ exposure to risk, enforces model citizenship, and preserves power relationships that curtail nurses’ capacity to have control over their work^27^ and ultimately their moral agency.

## Limitations

Several limitations are present in our study. The theoretical lens used was helpful in adding depth to the study, but this lens could be strengthened with more explicit elements attending to the sociopolitical conditions of nurses’ work that make care possible. In addition, this research was conducted in a Canada, a high-income country, and mainly in Ontario during the first wave of the pandemic, limiting the transferability of the findings. In comparison to many other places, resources were less scarce and case counts were lower likely having an impact on our participants’ experiences. Canada later experienced much higher waves of COVID-19 during 2021. While nurses may have gained confidence in their skills to provide care to patients, we speculate that nurses likely experienced even greater difficulties in their capacity to fulfill their ethical responsibilities of care over time given higher numbers of COVID-19 patients and growing fatigue.

## Conclusions

The pandemic has exacerbated the challenges nurses experience in providing good care to their patients. We discovered that infection control measures and priorities set in response to the pandemic made at distant population and organizational levels impacted nurses who continued to try to meet the ideals of care in close proximity to patients and their families. Despite the challenges that nurses encountered, the care they received themselves enabled them to continue to care for others. Nurses benefited most from the moral communities they had with their colleagues and occasionally nurse leaders, especially when they were supported in a face-to-face manner. We stress, however, that moral communities can only be sustained if nurses are afforded the working conditions that make it possible for them to support each other. Without care for nurses, care for patients will not be possible.

## References

[1] Iheduru-Anderson K. Reflections on the lived experience of working with limited personal protective equipment during the COVID-19 crisis. Nurs Inquiry 2021; 28(1): e12382. 10.1111/nin.12382 PMC7646033 33010197

[2] Rezee N Mardani-Hamoole M Seraji M. Nurses’ perception of ethical challenge in caring for patients with COVID-19: a qualitative analysis. J Med Hist Med 2020; 13: 23. 10.18502/jmehm.v13i23.4954 PMC8141204 34055239

[3] Sperling D. Ethical dilemmas, perceived risk, and motivation among nurses during the COVID-19 pandemic. Nurs Ethics 2021; 28(1): 9–22. 33000673 10.1177/0969733020956376 PMC7533465

[4] Jia Y Chen O Xiao Z, et al. Nurses' ethical challenges caring for people with COVID-19: a qualitative study. Nurs Ethics 2021; 28(1): 33–45. 32856534 10.1177/0969733020944453 PMC7653013

[5] Catania G Zanini M Hayter M, et al. Lessons from Italian front‐line nurses’ experiences during the COVID‐19 pandemic: a qualitative descriptive study. J Nurs Management 2021; 29(3): 404–411. 10.1111/jonm.13194 33107657

[6] Arnetz JE Goetz CM Arnetz BB, et al. Nurse reports of stressful situations during the COVID-19 pandemic: qualitative analysis of survey responses. Int J Environ Res Public Health 2020; 17(21): 8126. 10.3390/ijerph17218126 PMC7663126 33153198

[7] Gastmans C Schotsmans P Dierckx de Casterle B. Nursing considered as moral practice: a philosophical-ethical interpretation of nursing. Kennedy Inst Ethics J 1998; 8(1): 43–69. 11656753 10.1353/ken.1998.0002

[8] Vanlaere L Gastmans C. To be is to care: a philosophical-ethical analysis of care with a view from nursing. In: Leget C Gastmans C Verkerk M (eds). Care, compassion and recognition: an ethical discussion. Herent: Peeters; 2011, pp. 15–31.

[9] Peter E Simmonds A Liaschenko J. Nurses' narratives of moral identity: making a difference and reciprocal holding. Nurs Ethics 2018; 25(3): 324–334. 27220717 10.1177/0969733016648206

[10] Nortvedt P. Needs, closeness and responsibilities. An inquiry into some rival moral considerations in nursing care. Nurs Philos 2001; 2: 112–121.

[11] Tronto JC. Moral Boundaries: A Political Argument for an Ethic of Care. New York: Routledge, 1993.

[12] Kahlke R. Generic qualitative approaches: pitfalls and benefits of methodological mixology. Int J Qual Meth 2012; 13: 37–52.

[13] Reeves S Albert M Kuper A, et al. Why use theories in qualitative research? BMJ (Clinical Research ed.) 2008; 337: 631–634. 10.1136/bmj.a949 18687730

[14] Percy WH Kostere K Kostere S. Generic qualitative research in psychology. Qual Rep 2015; 20(2): 76–85.

[15] Eakin JM Gladstone B. “Value-adding” analysis: doing more with qualitative data. Int J Qual Methods 2020; 19: 1–13.

[16] Killackey T. Advance Care Planning in Advanced Heart Failure: A Relational Exploration of Autonomy. Canada: University of TorontoProQuest Dissertations Publishing, 2020, p. 28001811.

[17] Patton MQ. Enhancing the quality and credibility of qualitative analysis. Health Services Research 1999; 34(5): 1189–1208. 10591279 PMC1089059

[18] Hall WA Callery P. Enhancing the rigor of grounded theory: incorporating reflexivity and relationality. Qual Health Res 2001; 11(2): 257–272. 11221119 10.1177/104973201129119082

[19] Smith M Upshur R. Pandemic disease, public health, and ethics. In: Mastroianni AC Kahn JP Kass NE (eds). The Oxford handbook of public health ethics. New York, NY: Oxford University Press; 2019, pp. 1–19.

[20] Gilligan C. A Different Voice. Psychological Theory and Women's Development. Cambridge, MA: Harvard University Press, 1982.. In

[21] Lapum J Nguyen M Fredericks S, et al. “Goodbye … through a glass door”: emotional experiences of working in COVID-19 acute care hospital environments. Can J Nurs Res 2021; 53(1): 5–15. 33342299 10.1177/0844562120982420 PMC7754157

[22] Jameton A. Nursing Practice: The Ethical Issues. Englewood Cliffs: Prentice-Hall, 1984.

[23] Traudt T Liaschenko J Peden-McAlpine C. Moral agency, moral imagination, and moral community: antidotes to moral distress. The J Clinical Ethics 2016; 27(3): 201–213. 27658275

[24] Liaschenko J Peter E. Fostering nurses’ moral agency and moral identity: the importance of moral community. Hastings Cent Rep 2016; 46(5): S18–S21. 27649913 10.1002/hast.626

[25] Liu YE Zhai ZC Han YH, et al. Experiences of front‐line nurses combating coronavirus disease‐2019 in China: a qualitative analysis. Public Health Nurs 2020; 37(5): 757–763. 32677072 10.1111/phn.12768 PMC7405388

[26] Sheng Q Zhang X Wang X, et al. The influence of experiences of involvement in the COVID‐19 rescue task on the professional identity among Chinese nurses: a qualitative study. J Nurs Management 2020; 28(7): 1662–1669. 10.1111/jonm.13122 PMC7436396 32770772

[27] Mohammed S Peter E Killackey T, et al. The “nurse as hero” discourse in the COVID-19 pandemic: a poststructural discourse analysis. Int J Nurs Stud 2021; 117: 103887. 33556905 10.1016/j.ijnurstu.2021.103887 PMC9749900

